# Growth of *Caiman crocodilus yacare* in the Brazilian Pantanal

**DOI:** 10.1371/journal.pone.0089363

**Published:** 2014-02-28

**Authors:** Zilca Campos, Guilherme Mourão, Marcos Coutinho, William E. Magnusson

**Affiliations:** 1 Embrapa Pantanal, Corumbá, Mato Grosso do Sul, Brazil; 2 Instituto Chico Mendes de Conservação da Biodiversidade/Base RAN, Lagoa Santa, Minas Gerais, Brazil; 3 Coordenação de Biodiversidade, Instituto Nacional de Pesquisas da Amazônia, Manaus, Amazonas, Brazil; University of Calgary, Canada

## Abstract

We studied growth of the caiman, *Caiman crocodilus yacare*, in the Brazilian Pantanal for 27 years between 1987 and 2013.We recaptured 647 of 7769 *C. c. yacare* initially marked in an area of 50 km^2^, in two ranches. We were able to determine size at age accurately for 24 male and17 female caimans that had been marked at hatching or less than 1 year old, and recaptured over periods of 5 to 24 years. The other 606 caimans were used to evaluate short-term growth rates. Age-size relationships were estimated using growth models from the Richards family of curves (full model, von Bertalanffy and monomolecular). The form of the relationships differed between analyses based on caimans of known age and analyses based on integration of growth rate on size relationships for caimans whose ages were not known. Individuals showed large variation in short-term growth rates, but data on known-age animals indicated little between-individual variability in long-term growth rates. There was evidence of a small effect of rainfall, but not temperature, on short-term growth of small caimans, but most variation in growth rates was unexplained by variables other than age and sex. Data on known-age individuals indicated that female *C. c. yacare* generally reach sexual maturity between 10 and 15 years of age. Because of the asymptotic relationship between age and size, deviations of observations from the model for age are larger than for size, and estimates of age at a given size have greater errors than estimates of size at a given age. Integration of growth rate on size relationships may be adequate for estimating size from age in many cases, but accurate estimates of age from size require data on known-age individuals over the size range of the species.

## Introduction

Information on growth is important for many demographic models used in conservation biology and wildlife management. However, it is extremely difficult to determine size-age relationships of wild crocodilians directly because they are long-lived, wary, and may disperse dozens or hundreds of kilometres between hatching and reaching reproductive maturity [1]–[4].

Size-age relationships of many organisms are sigmoidal, with initially slow increases in size with age followed by a period of fast growth, and ending near an asymptote when there is little or no measurable increase in size with increase in age. In the absence of measurements of individuals with known ages, the relationship between size and age can be estimated from data on many individuals which were captured twice, giving two sizes and a known interval between measurements for each animal. Most researchers use the latter strategy, and try to establish size-age relationships by integrating data on short-term growth rates of individuals of different sizes [5]. However, these methods are controversial [6]–[10]. While there are good theoretical or practical reasons to use different models, choosing from them is difficult because there are limited data on known-age animals for validation.

Most biologists have used models from the Richards family of curves to investigate growth in reptiles [11], [12], [8], but crocodilians may show growth trajectories soon after hatching that do not conform well to the Richards sigmoidal model. During the first year, hatchlings often show growth rates that fluctuate between very high and very low values, [2], [13], [14], and these are not predicted by the sigmoidal models. As large deviations from the trajectories predicted by the models from the Richards family of curves only occur in the first year or so after hatching, they do not greatly affect estimates of age based on size [13]. These models differ only in the parameter m that defines the shape of the Richards curve, but this greatly affects estimates of age at a given size.

Analyses of growth rate on size may allow evaluation of environmental restrictions on growth [15]. Seasonal and interannual variation in temperature and rainfall influence growth rates of crocodilians [14], [15], and the Pantanal is strongly seasonal, with a dry and sometimes cold winter, and a warm wet summer with extensive flooding. Therefore, we expected much of the inter-individual variation in growth rates of caimans to be related to the mean temperature and rainfall during the period over which growth was measured.

One of the principle problems with construction of size-age relationships based on relationships between size and growth rate is that individuals may have parallel growth trajectories [2], [13], [8] determined by genetic or environmental effects that were fixed early in development. Therefore, variation around the mean growth rate may not represent random or short-term environmental effects. When individuals have parallel growth trajectories, complex models with parameters for individuals may be appropriate [9]. It has been suggested that such models should not be used if individual variation in growth rate over time is much greater than variation among individuals [16], but this can only be determined if there are sufficient individuals with multiple recaptures, which is an uncommon situation in crocodilian studies.

Very complex analyses have also been used to determine the mean size at hatching based on morphological relationships of larger individuals [9], but errors in assigning size at hatching probably have little effect on estimates of the overall form of the size-age relationship, or parameters estimated from it, because differences in estimates of hatching size differ only by a few cm [16].

In this study, we use a unique set of capture-recapture data for *C. c. yacare* to describe growth of the species in the Brazilian Pantanal, and to evaluate bias in estimates of age based on growth rates of different sized individuals that were not initially captured soon after hatching. Pantanal caimans are sometimes considered a separate species (*Caiman yacare*), but there is continuous genetic and morphological variation between individuals from the Pantanal (*Caiman crocodilus yacare*) and Amazônia (*C. c. crocodilus*), and recognition of the species would require an arbitrary geographic boundary between the taxa. A long-term capture-recapture study provided sizes of dozens of known-age individuals up to 25 years old and shorter-term capture-recapture data on hundreds of other individuals of all sizes. We had data on sizes of males and females at hatching, and large animals that had essentially stopped growing, which allowed us to parameterize the models throughout the whole life cycle.

This extensive data set for known-age individuals allowed us to accurately describe the size-age relationship for the species in our study area using the full Richards model. However, use of these data for comparisons with other studies may not be valid because most other researchers have not had access to data on known-age individuals in the field. We therefore compared the size-age sigmoidal curves based on known-age individuals and using the full Richards model with (1) curves derived from the full Richards model and data on recaptures of individuals whose ages were unknown, (2) curves based on the von Bertalanffy model for individuals without known ages, and (3) curves based on the monomolecular model for individuals without known ages. This allowed us to evaluate to what extent model choice and data type affect estimates of age based on size, and size based on age.

## Materials and Methods

We studied caiman on two ranches in the Nhecolândia region of the Brazilian Pantanal between 1987 and 2013. Nhumirim Ranch (18°59′S, 56°39′S) has an area of 4,310 ha containing about 100 ephemeral lakes. Campo Dora Ranch (18°55′S, 56°40′W) covers about 40,000 ha, including stretches of two intermittent rivers and seasonally flooded grasslands [3].

We captured caimans during the night with nooses or by hand in the lakes and intermittent rivers, and during the day when females were guarding nests or the caimans left pools during terrestrial activity [17]. We marked caimans individually with numbered plastic tags placed in the raised single tail scutes, aluminium numbered tags attached to one of the interdigital membranes of the left hind leg, and/or by removing single and double tail scutes in unique combinations. To measure snout-vent length (SVL-cm), we placed the caiman on its back and measured the distance from the tip of the snout to the end of the cloacal scales with a measuring tape (limit of reading 0.1 cm). We identified the sex of caimans from the presence of penis or clitoris. We could not confidently determine the sex of juveniles under 30 cm SVL and they were recorded as undetermined unless they were recaptured later at a larger size. We had used surgery to determine the sex of 25 male and 41 female hatchlings from 6 nests in a previous study [18], and used their sizes to represent size at hatching in the growth models.

We estimated growth rate as the difference in SVL between captures divided by the time interval between captures, and used geometric mean SVL to illustrate the relationship between growth rate and size. Age of juveniles up to 30 cm SVL was estimated as the interval between the month of capture and April, which is the month in which most individuals hatch in this area [18]. Errors in age for animals estimated to be less than one year old are unlikely to be greater than 1 month.

We used nonlinear estimation of the parameters for the Richards model [11] in the SYSTAT version 8 program to describe the relationship between size and age. The Richards model is *L_R_* =  [*A* ^(1−*m*)^−(*A* ^(1−*m*)^−(*A* ^(1−*m*)^−*L_c_* ^(1−*m*)^) exp (−2*t*/*T* (*m*+1))] ^1/(1−*m*)^, where L_R_ and L_C_ are SVL of animals at recapture and at first capture, respectively. A is the theoretical mean maximum SVL for individuals of the species, T is an index of the time necessary to approach asymptotic size, t is the interval between capture and recapture, and m is a parameter describing the shape of the curve [19]. For specific models, we fixed the parameter m at 0.667 (von Bertalanffy) or zero (monomolecular). For some models, the asymptotic-size parameter (A) was fixed at the mean size of the ten largest individuals [8].

Seasonal variation in temperature and rainfall is high in the study area. Summers are hot and wet, with extensive flooding, and winters are dry with both high and low temperatures [18]. Mean monthly temperature and rainfall in the months between capture and recapture were used as predictor variables in multiple-regression models. Temperature and rainfall data for this study were obtained from the Nhumirim Meteorological Station [20]–[22], Nhumirim Ranch.

The research project was approved by the Brazilian Environmental Agency (IBAMA permit N^o^. 017/02) and by the Chico Mendes Institute for Biodiversity Conservation (ICMBio permanent license N^o^. 13048-1) for capture and marking of caimans (relevant legislation: IN N^o^. 154/2007). All procedures followed ethical practices for animals recommended by the Brazilian Agricultural Research Organization (Embrapa). Nhumirim Ranch is owned by Embrapa. Campo Dora is a private ranch, and its owners, Luis Gomes da Silva and family, have authorized caiman research since 1987.

## Results

All 6 nests we studied produced both males and females, but the proportion varied between nests. The mean snout-vent length (cm) of female hatchlings (mean  = 12.49, 95% CI  = 12.33–12.65) differed significantly (Student's t_64_ = 2.77, P = 0.007) from that of males (mean  = 12.84, 95% CI  = 12.65–13.03), but the difference was small.

**Figure 1:**
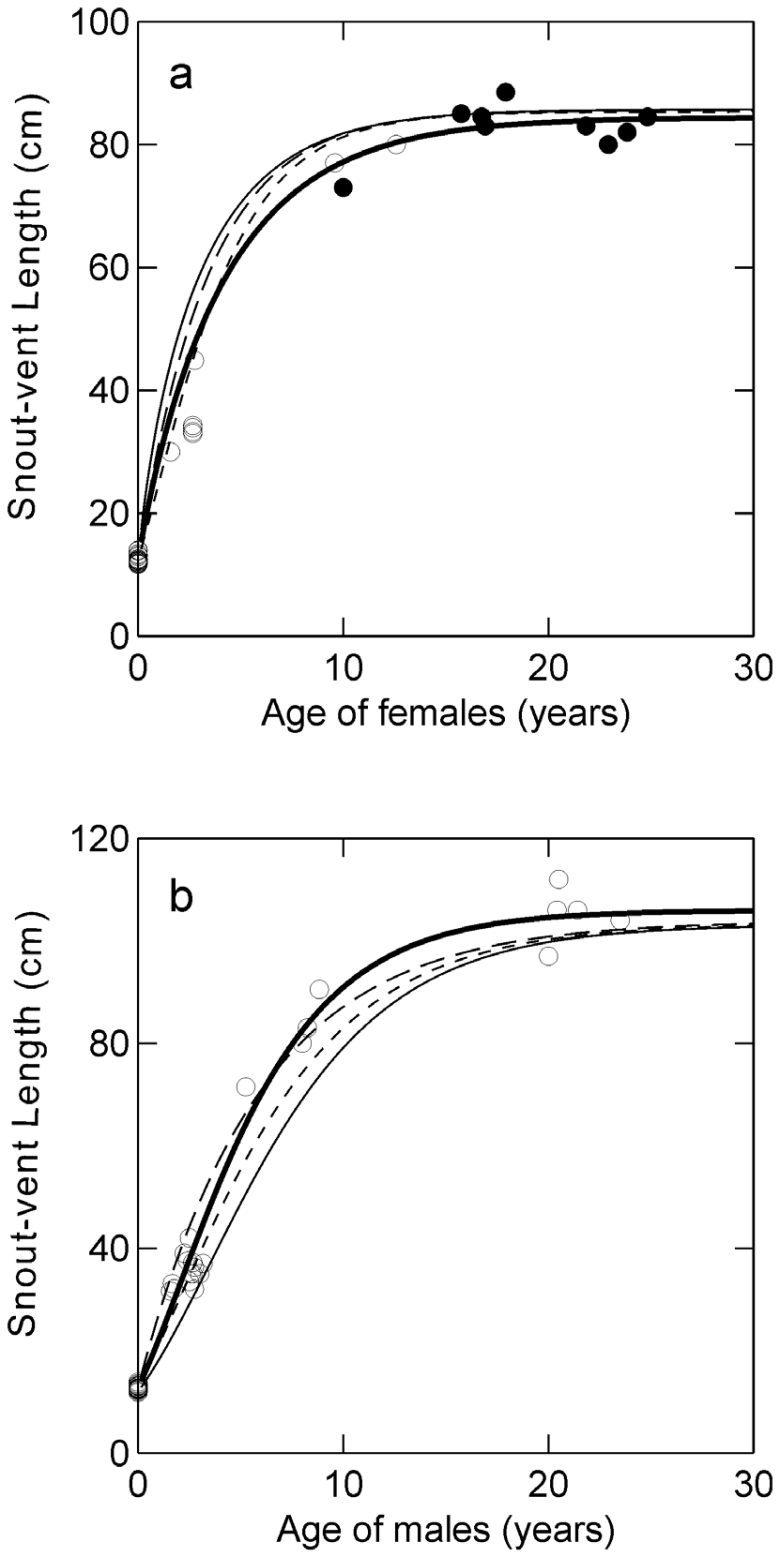
Relationship between size and age for known-age female (a) and male (b) *Caiman crocodilus yacare* (circles) based on the Richards curve (thick continuous line). Filled circles indicate females that were captured attending nests. Models based on growth-rate-on-size relationships for animals of unknown age are given by the fine continuous line (full Richards model), short dashes (von Bertalanffy, m = 0.667) and long dashes (monomolecular, m = 0).

Besides the hatchlings with age zero (age at hatching), ages of 24 males and 17 females could be estimated accurately (to within about 1 month) because they were first captured at SVL<14.0 cm. The full Richards model gave a good fit to these known-age animals (Figure 1: thick line) for both males (Asymptotic SVL  = 105.96, m = 0.778, T = 14.71) and females (Asymptotic SVL  = 85.73, m = 0.466, T = 4.02). The 95% confidence intervals for estimates of m from the full Richards model for both males (−1.699 to 1.350) and females (−0.572 to 2.129) were wide, and would include the values of m for both the monomolecular and von Bertalanffy models. Therefore, it would not be possible to statistically distinguish between the three models based only on data from caimans that did not have known ages.

Nine of the known-age females were captured beside nests and had therefore presumably reached sexual maturity. Sizes and ages of these individuals were close to those predicted by the full Richards model, and all but one of these had SVL ≥80 cm (Figure 1a: closed circles).

**Figure 2:**
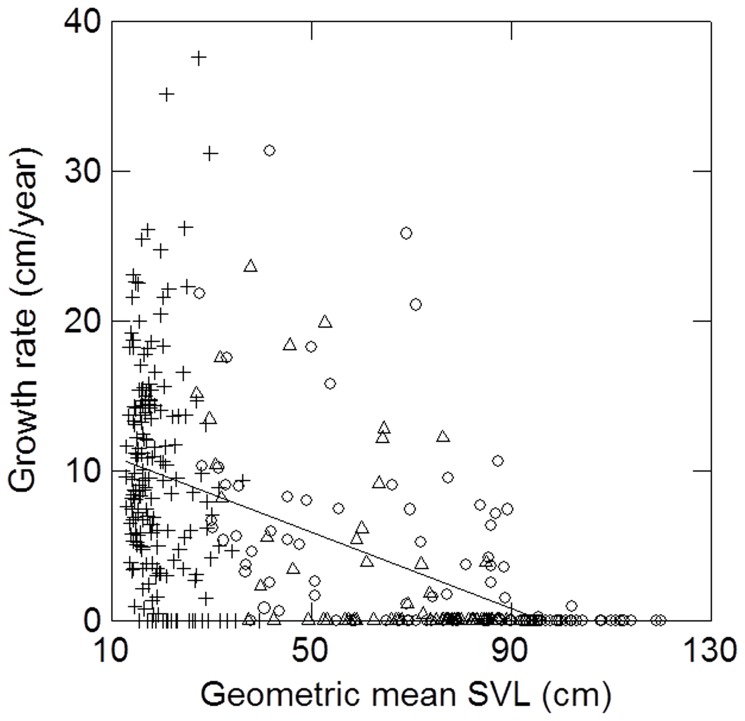
Relationship between growth rate and geometric-mean snout-vent length (between capture and recapture) of (+) juvenile, (Δ) female and (○) male *Caiman crocodilus yacare* in the Brazilian Pantanal.

Growth rates of individuals (GR - cm/year) varied greatly at all sizes (Figure 2). Multiple regression across all sizes for animals recaptured over intervals less than 2 years indicated effects of geometric-mean snout-vent length (SVL: P<0.00001) and sex (SX: P = 0.060), but there was little evidence for an effect of mean rainfall (RA: P = 0.380) or mean temperature (TE: P = 0.607) during the growth interval. Sex was entered as a dummy variable (0, 1). Restricting the growth interval to less than 1 year or analysing without restriction on the growth interval resulted in qualitatively similar results. However, the relationships apparently differed between large and small individuals. Multiple regression for individuals with SVL <50 cm when recaptured (GR  = 19.59 – 0.254*SVL + 1.044*RA + - 0.251*TE – 0.340*SX, N = 251, R^2^ = 0.098, P<0.00004) indicated significant effects of SVL (P = 0.00001) and rainfall (P = 0.004), but not of temperature (P = 0.452) or sex (P = 0.618). However, most individuals in this size class did not have their sex determined by surgery or subsequent recapture. Although the effect of rainfall was statistically significant, removal of this variable only reduced the variance explained by about 3% (R^2^ = 0.067).

Multiple regression for individuals recaptured over intervals less than 2 years with SVL >50 cm when recaptured (GR  = 9.87–0.132*SVL –0.184*RA + 0.196*TE – 0.605*SX, N = 205, R^2^ = 0.198, P<0.00001) indicated significant effects of SVL (P<0.00001) and sex (0.005), but not of rainfall (P = 0.194) or temperature (P = 0.291).

The full Richards model based on animals that had not been captured at hatching, and therefore did not have known ages (Figure 1b: fine continuous lines), underestimated the sizes of known-age individuals for males and overestimated sizes for females (Figure 1a, circles and thick continuous line). Fixing the growth parameter (m) to 0.667 (von Bertalanffy model) only slightly improved the fit for males, but the von Bertalanffy model was an improvement over the full model for females (Figure 1a, short dashes). Fixing the growth parameter (m) to zero (monomolecular model) only slightly improved the fit for females, but was an improvement over the full model for males (Figure 1b, long dashes).

A potential source of error is the large number of animals with essentially zero growth rates (Figure 2) at sizes much smaller than the asymptote for known-age individuals (Figure 1). These animals must have died or, more likely, just were not recaptured later when they had returned to positive growth. Based on known-age individuals, the asymptote estimated from the growth over short intervals for males (Figure 1b: fine and broken lines) is too low based on data from known-age animals (Figure 1: points and thick line). However, fixing the Richards asymptote parameter (A) at the mean for the 10 largest individuals (mean  = 115.25, min  = 112, max  = 120, S = 2.64), which was higher than the asymptote estimated for known-age individuals (106 cm), only worsened the fit of the Richards, monomolecular and von Bertalanffy models based on animals of unknown age.

## Discussion

We detected effects of sex, size and weather on growth rates, and therefore on size-age relationships, but most of the variation in growth rates was unaccounted for. The choice of model and type of data had much greater effects on the size-age relationship and estimates of parameters derived from it, such as age at first reproduction, than ecological variables.

Growth trajectories of males and females were different, and those differences probably manifest early in development because males were significantly larger than females at hatching. We were unable to detect an effect of sex on growth rates of individuals <50 cm SVL at recapture. Although most individuals in that size range did not have their sex recorded, similar growth rates of males and females were also found for immature *Crocodylus johnstoni* [8].

There is debate as to whether crocodilians should be considered to have determinate or indeterminate growth [23], [24]. However, asymptotic growth models fit the data well for *C. c. yacare*, as they do for most crocodilians [8], suggesting that growth is determinate or growth of large animals is extremely slow. Males from the same region have sperm in penile grooves at about SVL  = 55.0 cm SVL, and males with SVL  = 90.0 cm have mature testes [25], which is much smaller than the asymptotic size (106 cm) based on known-age individuals. If reproduction affects growth in males it is presumably for behavioral rather than physiological reasons, and they appear to keep growing long after they are physiologically capable of reproduction.

Most of the females at or above the estimated mean asymptotic size (86 cm) were captured attending nests. Only one female less than 80 cm SVL and less than 15 years old was in attendance at a nest. This is in accordance with the sizes of nesting females in the area. Few nesting females are less than 80 cm SVL [26]. There may large differences in sizes of nesting females because they have different asymptotic sizes and grow little after reproduction, or because they start reproducing well before reaching asymptotic size. The evidence is sparse, but indicates that females may continue to grow, even if they start nesting with SVL<80 cm.

The large variation in growth rates for a given size was surprising. At any given size, some individuals had growth rates twice the mean, and some individuals had no detectable growth. Individuals must vary greatly in growth rate over time, because individuals with consistently high or low growth rates would have age-specific sizes very different from the mean, which was not the case for our known-age individuals. The high variation is unlikely to be due to measurement errors or errors in identification of individuals because these sources of error would also have affected estimates for known-age individuals. Errors of identification would also result in as many negative growth rates as positive growth rates, but no negative growth rates were recorded, except for very large individuals that had essentially stopped growing.

Growth rates of crocodilians are believed to be related to temperature [27], [28], and food resources may be related to rainfall, which induces flooding. However, we were unable to detect an effect of mean temperature, and only about 3% of the variation in growth rate was attributable to mean rainfall during the growth period, and only for individuals with SVL<50 cm at recapture. *C. c. yacare* is extremely social [17], [29] and growth rates may depend on the social milieu in which the individual finds itself. Also, diseases could affect growth rates, but we have no data on these factors.

Selecting fixed-shape models, such as the von Bertalanffy or monomolecular (e.g. [30], [31], [28], [8], [9]) does not appear to be a useful strategy. The Richards shape parameter did not conform to any of the standard models for male or female *C. c. yacare* (this study) or for *Alligator mississippiensis* [6]. The shape parameter also varies within sexes of *A. mississippiensis* between individuals raised in captivity or in the wild [6]. Despite the reasonable fit of some of the reduced models for a given sex, the variation between studies, and between males and females, makes it difficult to predict which should be used unless there is extensive data on known-age individuals, which would obviate the necessity to estimate size-age relationships from growth-rate on size.

Very short intervals between captures increase the effect of measurement errors, and very long periods reduce the variation between individuals [5]. Given that individuals may have consistently different growth rates [2], [13], [8] and the extreme variation in growth rates, even when very short (<30 and <100 days) and long (>365 and >730 days) periods were excluded, it could be questioned as to whether it is worthwhile to try to reconstruct size-age relationships based on growth-rate-on-size relationships. This probably depends on the objectives of the study. Estimation of mean sizes of animals of a given sex at a given age can probably done with reasonable accuracy (to within 10%) by any of the models we used, except those in which we attempted to fix asymptotic size. This may be adequate for estimating numbers of animals in different size classes at times after interventions, such as hunting. However, many growth studies are carried out to estimate the minimum age at reproduction (e.g. [1], [9], [32], [14]). Estimates of extremes, such as minimum and maximum, are generally not precise [33], but even more useful parameters, such as the age at which we are confident that 90% of individuals are reproducing, would be hard to estimate from growth curves based on capture-recapture data for animals without known ages. Most nesting females in this area have SVL >80 cm [26]. All of the models based on growth rates indicate that they would reach this size at about 9 years old, but the data on known-age individuals indicate that they reach this size at about 13 years of age, a difference of 30%.

Despite the great variation in growth rates of individuals throughout their lifetime, there was comparatively little inter-individual variation in age-size relationships for known-age individuals, and models based on growth-rate-on-size relationships may be adequate to estimate size at a given age. However, because of the curvature of the age-size relationship, estimates of age at a given size will be strongly dependent on the shape of the curve, which is reflected in the Richards parameter m. Small differences in estimation of the value of m will strongly affect estimates of age at a given size, and using a sample of the largest individuals captured [8] was not a substitute for estimates of asymptotic size based on known-age individuals. If precise estimates of age at first reproduction are necessary, there does not appear to be a viable alternative to the use of known-age individuals obtained by long-term studies or the use of other approaches, such as skeletochronology [34], [35].
